# The Yin and Yang of Autosomal Recessive Primary Microcephaly Genes: Insights from Neurogenesis and Carcinogenesis

**DOI:** 10.3390/ijms21051691

**Published:** 2020-03-01

**Authors:** Xiaokun Zhou, Yiqiang Zhi, Jurui Yu, Dan Xu

**Affiliations:** 1 College of Biological Science and Engineering, Institute of Life Sciences, Fuzhou University, Fuzhou 350108, China; xkzhou1216@163.com (X.Z.); zhiyiqiang@foxmail.com (Y.Z.); N190820032@fzu.edu.cn (J.Y.); 2 Fujian Key Laboratory of Molecular Neurology, Institute of Neuroscience, Fujian Medical University, Fuzhou 350005, China

**Keywords:** autosomal recessive primary microcephaly, neurogenesis, carcinogenesis, centrosome, cell cycle, cell apoptosis

## Abstract

The stem cells of neurogenesis and carcinogenesis share many properties, including proliferative rate, an extensive replicative potential, the potential to generate different cell types of a given tissue, and an ability to independently migrate to a damaged area. This is also evidenced by the common molecular principles regulating key processes associated with cell division and apoptosis. Autosomal recessive primary microcephaly (MCPH) is a neurogenic mitotic disorder that is characterized by decreased brain size and mental retardation. Until now, a total of 25 genes have been identified that are known to be associated with MCPH. The inactivation (yin) of most MCPH genes leads to neurogenesis defects, while the upregulation (yang) of some MCPH genes is associated with different kinds of carcinogenesis. Here, we try to summarize the roles of MCPH genes in these two diseases and explore the underlying mechanisms, which will help us to explore new, attractive approaches to targeting tumor cells that are resistant to the current therapies.

## 1. Introduction

Embryonic stem cells are considered pluripotent, meaning that they are capable of differentiating into multiple cell types and maintaining the ability to self-renew to produce more of the same type of stem cells. Differentiated cells originating from stem cells make up the tissues and organs of animals and plants. Neurogenesis is the process of generating neurons by neural stem cell (NSC) proliferation, neuron migration, and differentiation. Proper neurogenesis is fundamental for normal brain development [1]. Carcinogenesis defines the initiation of a tumor, or the process of transforming normal cells into cancer cells, which is determined by some factors regulating cell growth and division [2]. Cancer stem cells (CSCs) are a subpopulation of stem-like-cell properties commonly shared with normal tissue stem cells, including extensive self-renewal ability (symmetrical and asymmetrical) and differentiation capacity [3]. CSCs exhibit characteristics of both stem cells and cancer cells [4]. 

**Figure 1:**
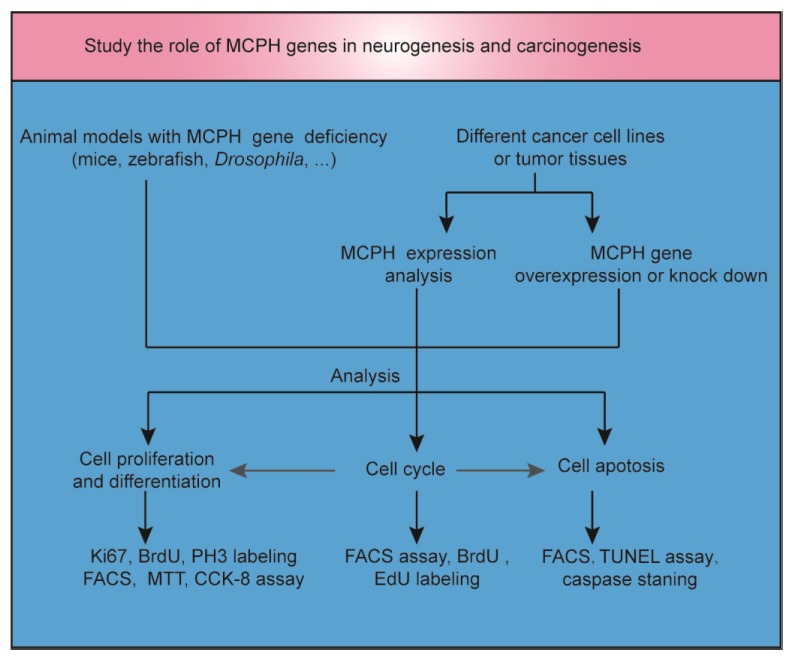
The role of MCPH genes in neurogenesis and carcinogenesis. Animal models with MCPH gene deficiency were established to study the role of MCPH genes in neurogenesis. MCPH gene overexpression or knockdown was induced in cancer cell lines or tumor tissues to study the role of MCPH genes in carcinogenesis. At the same time, MCPH gene/protein expression was analyzed by RNA sequencing, real-time PCR, or Western blot in carcinoma and precancerous tissue. Cell cycle, cell division, differentiation, and apoptosis were examined to study the mechanism of neurogenesis and carcinogenesis mediated by MCPH genes. BrdU, 5-bromo-2′-deoxyuridine; CCK-8, cell counting kit-8; EdU, 5-ethynyl-2′-deoxyuridine; FACS, fluorescent-activated cell sorting; MTT, 3-(4,5-dimethylthiazol-2-yl)-2,5-diphenyltetrazolium bromide; TUNEL, terminal deoxynucleotidyl transferase (TdT)-mediated dUTP nick end labeling.

Microcephaly, often described as ‘small head’, is a feature of many clinical disorders and can have environmental, maternal, or genetic etiologies. Autosomal recessive primary microcephaly (MCPH) is a rare encephalopathy caused by a dysfunction in neurodevelopment. To study the role of MCPH genes in neurogenesis and carcinogenesis, animal models and cancer cell lines or tumor tissues with MCPH gene deficiency or overexpression were established (Figure 1). Many genes linked to MCPH encode proteins involved in DNA repair or cell cycle control [5]. DNA replication, DNA repair, cell cycle progression, and the maintenance of genome stability are fundamental physiological processes that need to be tightly balanced to achieve normal neurogenesis in the brain or other tissues without resulting in carcinogenesis. Both neurogenesis and carcinogenesis have a gene expression signature that includes DNA and histone modifications [6,7]. In addition, there are overlapping migratory mechanisms between neural progenitor cells (NPCs) and brain tumor stem cells [8]. In light of the critical role of MCPH genes in mitosis, cell cycle, and apoptosis regulation, it is possible that the inhibition of the function of these genes may specifically affect the proliferation and survival of tumor cells [9]. In this review, we focus on novel insights into the overlapping mechanisms of neurogenesis and carcinogenesis by exploring the role of MCPH genes in brain development and cancer occurrence. Furthermore, we try to tap potential strategies for regulating the signaling of MCPH genes in cancer. 

## 2. MCPH Gene Deficiency Leads to Neurogenesis Defects 

MCPH patients show reduced brain size, pachygyria, and loss of the gray–white junction. Head circumference (HC) is one of the most useful indirect measurements for diagnosing microcephaly. Its clinical criterion is a HC that is three standard deviations below the mean (−3 SD) [10]. MCPH causes intellectual disability, accompanied by seizures, motor disorders, and speech and language disabilities [11]. The incidence of MCPH is about 2–12 in 10,000 live births [10]. At present, 25 genes have been identified and found to be associated with MCPH [12,13]. The cerebral cortex is the region most affected in microcephaly patients. During cortical development, MCPH genes function in several important early developmental events: NPC proliferation, differentiation into neurons, the migration of neurons to the specific position of the cortical plate, and the formation of axons and dendrites from which functional synapses are produced [14]. When any of these processes go awry because of a genetic mutation, the consequences can lead to severe neurological diseases such as MCPH.

To study the function of MCPH genes, animal models with MCPH gene mutation/deficiency have been established in zebrafish, *Drosophila*, mice, and non-human primates (cynomolgus macaque) [12,15]. Mimicking human MCPH symptoms, the most striking phenotype of MCPH gene deficiency mice was their significantly small brain size [13]. NPC (Pax6, Sox2, and Tbr2) and neuron (Tuj1, NeuN, Tbr1, and so on) markers were applied to test the effect of MCPH gene disturbance on cortical development. Fewer cells being labeled with proliferation markers Ki67 and 5-bromo-2′-deoxyuridine (BrdU) in MCPH gene-deficient mice brains indicates less NPC proliferation compared with wild-type mice [16,17,18]. NPCs increase the progenitor cell pool through symmetrical division (maintaining the progenitor cell pool) and produce progenitor cells and nerve precursors through asymmetrical division. The latter eventually undergo migration and differentiation to form the brain [19]. Some MCPH gene knockout (KO) mice showed a disrupted balance between symmetric and asymmetric division in the neocortex [16,20]. The neuronal migration defect was observed after *Mcph2* or *Mcph5* knockdown in the developing neocortex [18,21]. In addition, neuronal connectivity and neuron myelination defects were detected in MCPH gene KO mice [22,23]. 

## 3. Some MCPH Genes Can Be Considered as Potential Cancer Biomarkers 

Several MCPH genes, such as *MCPH1*, *MCPH2*, *MCPH5*, *MCPH7*, *MCPH8*, *MCPH10*, *MCPH12*, *MCPH15*, and *MCPH17-21*, have been reported to regulate both neurogenesis and carcinogenesis (Table 1). MCPH gene overexpression is always associated with centriole overduplication, multipolar spindles, anaphase-lagging chromosomes, and micronuclei. *MCPH4*, *MCPH7*, *MCPH8*, *MCPH10*, *MCPH13*, *MCPH17*, *MCPH19*, and *MCPH23* are highly expressed in different tumor tissues or cancer cell lines, indicating that some MCPH genes can be considered as oncogenes or potential cancer biomarkers [24,25,26,27,28,29,30,31]. The knockdown or inhibited expression of these genes may be a potential therapeutic method for cancer treatment [31,32,33]. In addition, the overexpression of *MCPH2*, *MCPH5*, *MCPH7*, *MCPH14*, *MCPH20*, *MCPH21*, *MCPH23*, and *MCPH24* is associated with aggressiveness and poor outcome in patients with cancer [28,34,35,36,37,38,39]. However, *MCPH1* and *MCPH15* are downregulated in tumor tissues or cancer cell lines and are considered as novel tumor suppressor genes [40,41]. 

**Figure 2:**
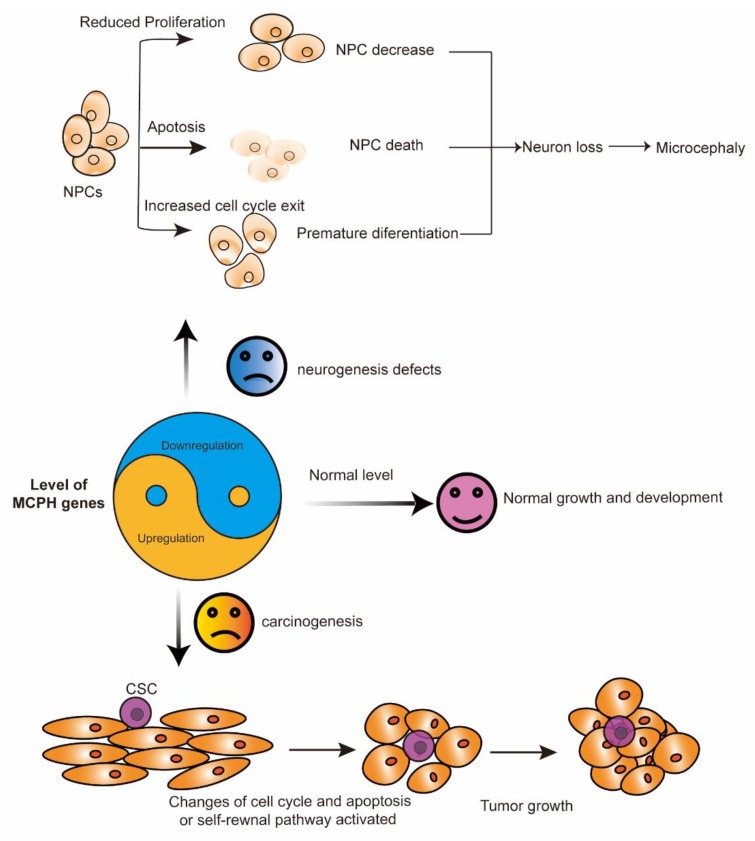
The yin and yang function of MCPH genes. MCPH gene deficiency (yin) leads to neurogenesis defects, while its overexpression (yang) is associated with carcinogenesis. In cortical NPCs, the loss of the MCPH gene results in premature differentiation, a reduction in cell proliferation, and increased apoptosis. These processes finally lead to neuron loss and microcephaly. During carcinogenesis, the overexpression of some MCPH genes, except for *MCPH1* and *MCPH15*, change the cell cycle and the cell apoptosis regulation of the cells and finally lead to tumor growth. CSC, cancer stem cell; MCPH, autosomal recessive primary microcephaly; NPCs, neural progenitor cells.

In summary, the expression level of the MCPH gene is important for normal brain development and that of tissues without cancer. In cortical NPCs, the loss of the MCPH gene results in cell division failure, a reduction in cell proliferation, and increased apoptosis. The most common phenotypes representing defects in neurogenesis include decreased neural progenitors, premature differentiation, and cell death. These processes finally lead to neuron loss and reduced brain size, which is also called microcephaly (Figure 2). The development of cancer is a complicated process in which a large number of factors interact to disrupt normal cell growth and division. Some MCPH gene upregulation is associated with aggressiveness and a poor outcome in patients with cancer, as indicated by the role of MCPH genes in carcinogenesis (Figure 2). 

## 4. The Centrosomal Root of MCPH Genes

The centrosome, which acts as the main microtubule-organizing center in animal cells as well as a regulator of cell cycle progression, is a non-membrane-bound organelle composed of two centrioles surrounded by pericentriolar material [91]. Centrosomes are closely linked to neurodevelopment, not only due to their key role in cell division but also because of their participation in cell polarization and migration in the developing brain [92]. Abnormal centrosomes are one cause of human tumors, whereas mutations in centrosome proteins such as MCPH genes have recently been genetically linked to microcephaly and dwarfism [93].

*MCPH1 (BRIT1)*, the first gene found to cause microcephaly, plays an important role in controlling mitosis, centrosome separation, and DNA damage repair [94]. *MCPH2* (*WDR62*), the next most common gene causative of microcephaly, showed cell cycle-dependent expression and functions in centriole biogenesis and mitotic spindle orientation [17]. MCPH3 (CDK5RAP2) protein products are important centrosome materials required for centrosome assembly and cell division [95]. MCPH5 (ASPM) participates in spindle localization during neurogenesis [96,97], while MCPH6 (CENPJ) serves as a centromere protein required for the spindle checkpoint [57,98]. In addition, MCPH6 regulates ciliary decomposition and neurogenesis through the KIF2A terminal directed motor protein [56]. It has been found that the TCP domain of MCPH7 (STIL), essential for the replication of the centriole, constitutes a proline recognition domain, forming a 1:1 complex with the short and highly conserved target motifs in MCPH7 [59]. This regulates the replication of centrioles in vivo [58]. Other studies have shown that the human microcephalic malformation protein rotatin (RTTN) directly interacts with STIL and acts downstream of STIL-mediated centrosome assembly [60], thereby affecting the development of the brain. *MCPH8 (CEP135)* is particularly important in the assembly, amplification, and microtubule binding of the centriole [99]. Mutations in this gene cause microtubule and centrosome assembly defects, which have a serious impact on the normal occurrence of nerves and cause primary microcephaly [63]. In the *Drosophila* nervous system, the central protein BLD10/MCPH8 is vital to the establishment of centrosome asymmetry in *Drosophila* neuroblasts [64]. In breast cancer, mutations in *MCPH8* promote centriole overduplication, leading to chromosome segregation errors in breast cancer cells [29]. MCPH9 (CEP152) acts as a scaffold and is essential for centriole expansion and spindle formation [100]. MCPH12 (CDK6), which encodes cyclin-dependent kinase 6, is associated with the centrosome during mitosis [101], and MCPH14 (SAS6) is a central component of centrioles and is necessary for their duplication and function [59,102].

Neurogenesis and carcinogenesis are both associated with abnormal centrosome duplications [61]. It is difficult to rationalize how centrosome anomalies lead to neurogenesis defects or tumors. Supernumerary centrosomes are sufficient to drive aneuploidy and the development of spontaneous tumors in multiple tissues [103]. Most MCPH genes, except *MCPH1* and *MCPH15*, are highly expressed in tumors, but there is a conspicuous absence of direct genetic evidence linking the level of the MCPH gene to supernumerary centrosomes in human carcinogenesis. Aneuploidy generated during development by centrosome amplification is not always compatible with tumor initiation. Supernumerary centrosomes are also reported in patients or mouse models with mutations in MCPH genes such as *MCPH3*, *MCPH7*, and *MCPH8* [63,104,105]. One explanation is that mammalian NPCs, compared with other cells, may be particularly vulnerable to centrosome amplification because they have to divide asymmetrically and, therefore, lead to premature differentiation or death [93]. Future work should study whether MCPH gene mutations result in tumors in other tissues apart from the brain. Another explanation of this discrepancy is that the mutations in the studied MCPH genes indicate that they are not null alleles. This is well studied in *MCPH7/STIL*. No expression or residual expression of MCPH7 causes an absence of centrosome or reduced centrosome, and the upregulation of STIL leads to centrosome amplification [61]. However, STIL mutations in patients leads to STIL stabilization and triggers centriole amplification [104]. Moreover, this discrepancy may be dependent on p53 expression. p53 is a tumor suppressor whose mutation is most commonly found in cancer cells. p53Plk4OE brains cause the accumulation of extra centrosomes and aneuploidy leads to premature neuronal differentiation, resulting in the generation of a microcephalic brain [92]. In contrast, supernumerary centrosomes in PLK4OE/p53cKO mice were sufficient to generate aneuploidy in the adult epidermis and accelerate skin tumorigenesis [106]. 

Both excessively high and overly weak expression of MCPH7 lead to microcephaly, through either reduced or amplified centrosome duplication [61]. Centrosome depletion or the disruption of its integrity also leads to neurogenesis defects or carcinogenesis. *MCPH6* deletion leads to a progressive loss of centrioles and centrosomes, which results in a substantial loss of neurons and microcephaly in the developing mouse brain [107]. The loss of the microcephaly proteins MCPH2 or MCPH5, or both, impairs centriole duplication and leads to centrosome and cilia loss [17]. In addition, centrosome loss also results in an unstable genome and malignant prostate tumors [108].

## 5. MCPH Gene Regulate Neurogenesis and Carcinogenesis through Regulation of Cell Cycle and Cell Division

**Figure 3:**
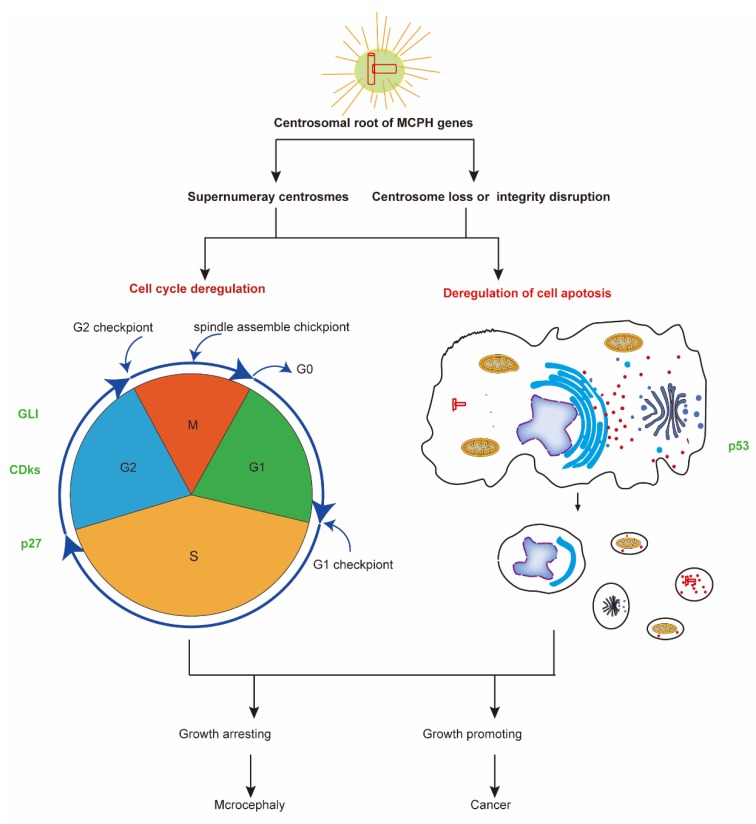
The common mechanism of neurogenesis and carcinogenesis. Most MCPH genes have a centrosomal root and take part in regulating the cell cycle and cell apoptosis. The deregulation of cell cycle and cell apoptosis processes leads to diseases including microcephaly and cancer. Some molecular factors, such as p53, CDK, and GlI family proteins, function in both MCPH-related neurogenesis and carcinogenesis processes. CDKs, cyclin-dependent kinases; GLI, human glioblastoma protein; MCPH, autosomal recessive primary microcephaly.

The centrosome is required for several cell cycle transitions, including G1 to S phase, G2 to mitosis, and metaphase to anaphase [109]. The centrosomal root of MCPH decides the role of MCPH genes in the regulation of cell cycle and cell division (Figure 3). Most MCPH gene deficiencies lead to reduced NPC proliferation in developing brains. This is evidenced by animal models with MCPH gene depletions. The Chk1–Cdc25–Cdk1 pathway is disrupted in *Mcph1*-deficient mice, which further distorts mitotic spindle alignment and shifts the division plane of neuroprogenitors [16]. Studies on *Mcph2* deficiency mice have indicated that MCPH2 mainly functions in neuron proliferation and differentiation by regulating neural stem cell division in the central nervous system [17,18,47,48]. The total number of cells and the thickness of the cortical plate were significantly decreased in *Mcph5* KO mice compared with wild-type mice. *Mcph5* deficiency leads to the abnormal proliferation and differentiation of nerve stem or progenitor cells and affects the development of the cerebral cortex [54]. Specifically, knockdown of *Mcph6* in mice brains by in utero electroporation suggests that MCPH6 regulates progenitor cell division by mediating the Ascl1-regulated generation of the central body and the stability of microtubules during neuronal mitosis [57]. As *MCPH12* is a key gene in cell cycle regulation, it can also regulate neuronal output and cortical size [66]. In addition, during the development of the hippocampus, p27 can negatively regulate the expression of MCPH12, thereby regulating the proliferation of hippocampal cells [68]. During the development of the neocortex, the loss of MCPH17 mainly affects neurogenic divisions [76]. In *Mcph20* mutant mice, BrdU labeling indicates decreased cell proliferation during the development of the cerebral cortex [22]. *MCPH25* depletion in a zebrafish model caused microcephaly and reduced neuron proliferation. At the end of cell division, MCPH25 and PLK1 partially co-localized to regulate cell division and control cell exfoliation [90]. 

Some MCPH-associated genes, such as *MCPH1*, *MCPH2*, *MCPH12*, *MCPH17*, and *MCPH20*, regulate both neuroprogenitor and cancer cell proliferation through regulation of the cell cycle and cell division. MCPH1 functions as a tumor suppressor. Overexpression of *MCPH1* inhibits human cervical cancer cell growth through regulating cell cycle-related proteins, such as cyclinA2/CDK2 and CDC25C-cyclinB/CDC2 [40]. Ectopic expression of *MCPH1* through genetic approaches effectively suppressed breast cancer cell proliferation and colony formation in vitro and tumor growth in vivo [110]. The knockdown of *MCPH2* induces G2/M phase arrest in gastric cancer (GC) [44]. In glioblastoma, SUMO1 modification stabilizes MCPH12 drives the cell cycle, resulting in the development and progression of cancer [69]. *MCPH17* is overexpressed in human colon cancer tissues and cell lines, and *MCPH17* knockdown represses cellular proliferation and colony formation [26]. *MCPH20* knockdown is known to suppress prostate cancer proliferation [36].

Though their role in neurogenesis has not yet been reported, the abnormal expression of most MCPH genes affects the cell cycle or cell proliferation in cancers. The *MCPH4* gene encodes a kinetochore protein that plays an important role in mitosis. The abnormal expression of *MCPH4* in colorectal tumors affects cell proliferation and apoptosis [52]. In GC cells, miR-193b-3p might contribute to the mitotic nuclear division of GC cells by mediating the upregulation of *MCPH4* [27]. *MCPH7* has a role to play in both primary microcephaly and cancer by its involvement in cell cycle perturbations and chromosomal segregation [61]. *MCPH7* regulates the proliferation of GC cells through the IGF-1/PI3K/Akt pathway. The deletion of this gene induces cell cycle arrest in the G2/M phase and induces GC cell apoptosis [32]. In prostate cancer (PCa), MCPH7 can affect the MAPK/ERK, PI3K/AKT, and AMPK signaling pathways, consequently promoting the proliferation of PCa cells through colony formation and inhibition of cell apoptosis [28]. MCPH13 is a human kinetochore protein. The overexpression of forkhead box M1 (FOXM1) promotes MCPH13 expression and proliferation in lung cancer cells [25]. In colorectal cancers, *MCPH14* gene mutations cause mitotic abnormalities and lead to cancer [73]. In non-small cell lung cancer cell lines and primary lung adenocarcinomas in different tissue types, the expression of *MCPH15* was strongly downregulated, affecting cell adhesion, migration, and regulation of the G1 cell cycle phase [41]. The *MCPH18* gene regulates ovarian cancer cell proliferation, migration, invasion, and epithelial mesenchymal transformation [78]. The *MCPH19* gene is associated with the occurrence of PCa and cholangiocarcinoma by regulating the G1 cell cycle phase and apoptotic pathway [82,84]. In colon cancer, the downregulation of *MCPH19* can induce cell cycle arrest in the G0/G1 or S phase [85], subsequently inhibiting the occurrence of cancer. In lung adenocarcinoma, the upregulation of Yap1 upregulates *MCPH19* expression, which inhibits cell apoptosis and promotes cell growth and tumorigenesis [83]. *MCPH20* can be used as a potential oncogene. In vivo human studies have found that *MCPH20* knockdown by small interfering RNA leads to G2 arrest and reduced proliferation in PCa cells. *MCPH20* knockdown also affects the cell cycle and regulates the expression of GAD45A, GAD45B, p21, PIDD, and SHISA5, all of which contribute to growth arrest and apoptosis induction [36]. In human colon cancer (CC), the KO of *MCPH23* can inhibit the proliferation, in vitro migration, and xenograft tumorigenesis of CC cells by inhibiting the cell cycle during G2/M phase transition, and inducing cell apoptosis [31]. In PCa, MCPH23 interacts with a variety of proteins during the regulation of PCa-associated cell cycles. These proteins include Aurora kinase A, Aurora kinase B, and cyclin-dependent kinase 1 [38]. Consistently, the deletion of low-density lipoprotein-related receptor 5 inhibits liver cancer cell proliferation via destabilizing MCPH24 in a β-catenin-independent way [111].

## 6. MCPH Gene Regulate Neurogenesis and Carcinogenesis via Cell Apoptosis Regulation

Apoptosis (or programmed cell death) is one of the central cellular processes in brain development and carcinogenesis. MCPH genes regulate proper centrosome formation, including the complex comprising the centrosome, centrioles, and connecting filaments that is required for apoptosis [112] (Figure 3). Caspase staining indicates more apoptosis in the *Mcph1*-null neocortex before and after γ-irradiation compared to wild-type samples [113]. *Mcph5* deficient inhibits postnatal cerebellar neurogenesis through apoptosis in mice brains [114]. In mammals, the loss of *MCPH17* leads to substantial cytokine depletion and apoptosis in neuronal progenitor and male germline cells, leading to severe microcephaly and testicular dysplasia [76]. In mammalian and *Drosophila* models, the loss of MCPH17 can additionally cause DNA damage accumulation and chromosomal instability. This leads to a failure of cell division and apoptosis of neural progenitor cells [77]. Used to mark apoptotic cells and associated with a missense variant, caspase 3 expression was increased in the cortex of *Mcph19* mutant mice during brain development [81]. *Mcph20* mutant mice showed increased numbers of apoptotic cells, as revealed by TUNEL staining in the cerebral cortex [22].

The downregulation of most MCPH genes leads to apoptosis, indicating that most of them can be considered as potential therapeutic targets for cancer treatment. Genetic deletion of *Mcph5* in mice reduces the growth of medulloblastoma and increases DNA damage [114]. Inducible knockdown of *MCPH7* in cancer cells in vitro decreased CDK1/CYCLIN B activity and induced apoptosis [115]. In medulloblastoma cell lines and mouse models, the absence or deletion of MCPH17 increased apoptosis, thus suggesting its potential role in medulloblastoma treatment [33]. In addition, the apoptosis rate was increased in *MCPH19* knockdown PCa cells [82]. *MCPH20* was consequently considered to be a novel candidate oncogene. The knockdown of *MCPH20* in DU145 and PC3 PCa cells induced cell apoptosis [36]. Consistently, high expression of *MCPH12* affects cell apoptosis by altering the cell cycle process, leading to the occurrence of various cancers [67,116]. In contrast, MCPH1 is an early DNA damage response protein [117]. In the process of carcinogenesis, the overexpression of *MCPH1* inhibits uncontrolled cell growth by regulating several apoptosis-related proteins and activating cell apoptosis. These include p53, Bcl-2, Bax, cytochrome c, caspase-3, and PARP-1 [40,118].

## 7. The Molecular Regulators of MCPH Genes during Neurogenesis and Carcinogenesis

Cell division and cell apoptosis regulation by MCPH genes indicate that there are common molecular mechanisms underlying the development of the brain and cancer during neurogenesis and carcinogenesis, respectively. Several cell signaling pathways regulated by MCPH genes have been studied. Some molecular factors, such as p53, CDK, and GlI family proteins, function in both neurogenesis and carcinogenesis processes (Table 2).

The p53 gene (TP53) is classified as a tumor suppressor gene, and upregulation of its activity led to neural progenitor apoptosis in MCPH gene-deficient mice. Deletion of *Mcph5* increases DNA damage and induces postnatal cerebellar progenitor apoptosis that depends on p53 [114]. *Mcph17* KO led to TP53 activation and the TP53-dependent apoptosis of NPCs and neurons in developing brains. NPC death was dramatically reduced in *Citk* and *Trp53* double KO mice [77]. During carcinogenesis, the overexpression of *MCPH1* inhibits the migration and invasion of lung cancer cells through the accumulation of p53 [42]. MCPH1 regulates p53 activity and protein stability through blocking MDM2-mediated p53 ubiquitination in breast cancer cells [110]. Conversely, some MCPH gene overexpression promotes cancer cell progression, sometimes through the regulation of p53 activity. MCPH12 contributes to tumor formation by inducing a complex transcriptional program to block p53 in hematopoietic cells [132]. Microarray and bioinformatics analyses indicate that the overexpression of the *MCPH17* gene in human colon cancer tissue can affect the occurrence of cancer through the p53 signaling pathway [26]. Consistently, inactivation of some MCPH genes typically inhibits cancer cell progression, sometimes by p53 activity regulation. *MCPH7* depletion induces cancer cell apoptosis in a p53-independent manner [115]. Transcriptome analysis by RNA sequencing demonstrated that *MCPH20* suppression led to transcriptional changes of genes involved in the p53 signaling pathway, which may lead to apoptosis in prostate cancer [36]. Thus, these results suggest that the p53 status must be considered when designing combinatorial or sequential approaches in precision medicine.

Emerging evidence suggests that tumor cells require specific interphase cyclin-dependent kinases (CDKs) for proliferation. Mutant *MCPH1* cells have low levels of Tyr 15-phosphorylated Cdk1 (pY15-Cdk1) in S and G2 phases, while Cdk1 triggers the translocation of MCPH7 from centrosomes to the cytoplasm [104,133]. In addition, *MCPH19* knockdown in human prostate carcinoma cells decreases CDK2 and CDK4 expression [82]. This indicates the role of CDKs in microcephaly and carcinoma. Cdkn1b (cyclin-dependent kinase inhibitor 1B), also known as p27, is involved in the regulation of the cell cycle and functions in both neurogenesis and carcinogenesis. During hippocampal neurogenesis, the proliferation of progenitor cells specifically relies on the p27-dependent regulation of MCPH12 kinase activity. CDK2, CDK4, and cyclin D1 were downregulated, whereas p21 (Waf1/Cip1) and p27 (Kip1) were upregulated in *MCPH19* knockdown cells compared with in control cells [68]. The role of p27 in carcinogenesis was revealed through studies involving MCPH19 [82]. In *MCPH20*-silenced hepatocellular carcinoma cells, the levels of cyclins E1, D1, and B1 were profoundly decreased, while the p27 protein level specifically increased [88].

The glioblastoma (GLI) zinc-finger transcription factors, acting as effectors of Sonic hedgehog (Shh) signaling, belong to the C2H2-type zinc finger protein subclass, which is critical for normal embryo development and cancer progression [134,135]. In dopaminergic neurons, MCPH7 functions through the Shh pathway by releasing the inhibition of tumor suppressor protein suppressor of fused (SUFU) to GLI1, and thereby enhances the Shh target gene transcription that is required for neural proliferation, protection, and regeneration [127]. During pancreatic carcinogenesis, MCPH7 is responsible for the depression of GLI1, which is a crucial step in activating Hh signaling in cancer cells [128]. GLI3 controls the onset of cortical neurogenesis by determining the level of MCPH12 expression [66]. Similarly, GLI2 binds to the MCPH12 promoter and activates *MCPH12* expression, thereby promoting uncontrolled medulloblastoma cell proliferation [129]. 

In addition, some MCPH genes function in pathways known to be important for stem cell self-renewal, such as the Wnt (*MCPH5* and *MCPH18*), Notch (*MCPH12*), Hippo (*MCPH3*, *MCPH19*, *MCPH20* and *MCPH24*), and Shh (*MCPH7* and *MCPH12*), all of which were previously identified as being relevant to cancer (Table 2). Hence, a clinical trial using inhibitors to block these pathways can be set for cancer treatment. 

## 8. Conclusions and Perspectives

During brain development, mitosis, the cell cycle, and genomic stability maintenance are particularly important. Disturbance of these processes will cause neurological disorders such as MCPH. Meanwhile, MCPH genes and related pathways play important roles in the process of carcinogenesis or the maintenance of CSCs. Since oncogenesis has always been a difficult problem to address, exploring the molecular mechanism of oncogenesis and finding solutions for it are currently an important research direction. Most importantly, the depletion of some MCPH genes typically suppresses cell division and tumor growth, indicating that the MCPH genes can be considered suitable targets in cancer treatment. Recently, because of its association with microcephaly and other severe neurological diseases, the Zika virus became an emerging flavivirus [136]. Interestingly, several studies have applied this virus to the treatment of glioblastoma—the most aggressive form of brain cancer [137,138,139]. Here, we explored the common role of MCPH gene in neurodevelopment and carcinogenesis. This not only helped in understanding the pathogenesis of microcephaly and cancer but also facilitated investigations into therapeutic strategies against each of these elements.

Traditional cancer therapies have focused on shrinking tumors, but with only moderate improvements. Recently, researchers have focused on studying how cancers arise and developing drugs that kill CSCs. The targeting of cancer by inactivating MCPH genes has the potential to be very attractive. Our study on MCPH genes provides many of the same features and factors shared by neurogenesis and carcinogenesis. However, the common mechanisms that underlie NSCs and CSCs still need to be explored. There are some studies relating MCPH genes with Wnt, Notch, Hippo, and Hedgehog signaling pathways, which are critical for stem cell self-renewal. In the future, substantial effort should be focused on determining whether MCPH genes regulate tumor growth through the regulation of CSC self-renewal. 

## Figures and Tables

**Table 1:** MCPH genes regulate both neurogenesis and carcinogenesis.

Gene	Role in/Effect on Neurogenesis	Ref.	Role in/Effect on Carcinogenesis	Ref.
*MCPH1 (BRIT1)*	Premature neurogenic production	[16]	Cell cycle and apoptosis (cervical cancer)	[40]
	Reductions in head circumference, premature chromosome condensation, and hypoplasia of the corpus callosum	[15]	Migration and invasion (lung cancer)	[42]
*MCPH2* *(WDR62)*	Mitotic spindle orientation	[43]	Cell cycle (gastric cancer, GC)	[44]
	Regulating intermediate neural and glial progenitors	[45]	Centrosome amplification (ovarian cancer, OC)	[46]
	Cell cycle, centriole biogenesis, and mitotic spindle orientation	[17]	Cell growth (lung adenocarcinoma)	[34]
	Regulating neural stem cell division	[47,48]		
*MCPH3 (CDK5RAP2)*	Reduced neurons and neural progenitors, premature cell cycle exit, and increased cell death	[49]	N/A (Leukemia)	[50]
	Premature neuronal differentiation	[51]		
*MCPH4 (CASC5)*	N/A		Cell proliferation and apoptosis (colorectal cancers, CRCs)	[52]
			Nuclear division of cells (GC)	[27]
*MCPH5* *(ASPM)*	Defects in cortical layers IV, V, and VI formation and imbalance of horizontal and vertical neurites	[53]	Differentiation and metastasis (bladder cancer)	[35]
	Abnormal proliferation and differentiation of nerve stem/progenitor cells	[54]	Cell cycle progression (pancreatic cancer prognosis)	[55]
*MCPH6* *(CENPJ)*	Cell proliferation, cell apoptosis	[56]	N/A	
	Centrosome generation and microtubule stability, progenitor division, and neuronal migration	[57]		
*MCPH7* *(STIL)*	Centriole duplication	[58,59]	Cell proliferation, cell cycle G2/M phase and apoptosis (GC)	[32]
	Centromere assembly	[60]	Cell proliferation and apoptosis (prostate cancer, PCa)	[28]
	Cell cycle and chromosomal segregation	[61]	DNA repair (OC)	[62]
*MCPH8 (CEP135)*	Assembly of centrosome and microtubule	[63]	Centriole duplication (breast cancer)	[29]
	Establishment of centrosome asymmetry	[64]		
*MCPH10 (ZNF335)*	Neural progenitor self-renewal, neurogenesis, and neuronal differentiation	[65]	tumor progression (Bladder cancer)	[24]
*MCPH12* *(CDK6)*	Regulating radial glial cells G1 and S phases	[66]	DNA damage response, apoptosis (epithelial ovarian cancer)	[67]
	Positively regulates the proliferation of hippocampal progenitors	[68]	Cell cycle (glioblastoma)	[69]
	Proliferation of neural stem cells	[70]	Cell cycle, cell proliferation and angiogenesis (hematopoietic malignancies)	[71]
			Cell cycle and apoptosis (T-cell acute lymphoblastic leukemia)	[72]
*MCPH13 (CENPE)*	N/A		Cell G2/M phase and proliferation (lung cancer)	[25]
*MCPH14 (SAS6)*	N/A		Centrosome amplification, mitotic abnormality (CRCs)	[73]
*MCPH15 (MFSD2A)*	Blood–brain barrier disruption	[74]	Cell cycle (G1 phase) and matrix attachment (lung cancer)	[41]
*MCPH16* *(ANKLE2)*	Reduced cell proliferation	[75]	N/A	
*MCPH17* *(CIT)*	Apoptosis of neuronal progenitors [76,77]		Cell cycle and apoptosis (colon cancer, CC)	[26]
			DNA damage, proliferation, cell senescence and apoptosis (medulloblastoma)	[33]
*MCPH18* *(WDFY3)*	Perinatal lethality, telencephalic junction, axonal connectivity defect, and localization of glial guidepost cells	[23]	Cell proliferation, migration, invasion, and epithelial-to-mesenchymal transition (OC)	[78]
	Neurodevelopmental delay, intellectual disability, macrocephaly, and psychiatric disorders	[79]		
	Distribution in neuronal projection and axon guidance	[80]		
*MCPH19* *(COPB2)*	Increased apoptosis in the brain and slow growth of neurospheres	[81]	Cell cycle, apoptosis and proliferation (PCa)	[30,82]
			Cell proliferation and apoptosis (lung adenocarcinoma)	[83]
			Cell G1 phase, proliferation and apoptosis (cholangiocellular cancers)	[84]
			Suppresses cell proliferation and induces cell G0/G1 or S phase arrest (CC)	[85]
			Cell proliferation and apoptosis (GC)	[86]
*MCPH20* *(KIF14)*	Flat head, motor impairment, growth retardation, decreased cell proliferation, and cell death	[22]	Induced cell cycle arrest and apoptosis (PCa)	[36]
			Regulation of the expression of transcription factors (SP1, YY1) (ovarian cancers)	[87]
			Cell cycle and proliferation (hepatocellular carcinoma)	[88]
*MCPH21* *(NCAPD2)*	Reduced cortex	[89]	Promote cell cycle and enhance [39] invasion (Triple-negative breast cancer)	
*MCPH23* *(NCAPH)*	N/A		Cell cycle (G2/M phase), cells proliferation, migration and apoptosis (CC)	[31]
			Cell cycle (PCa)	[38]
*MCPH24* *(NUP37)*	N/A		Cell growth, migration and invasion (hepatocellular carcinoma)	[37]
*MCPH25* *(MAP11)*	Microcephaly, decreased neuronal proliferation, a reduction in white matter, and hypoplasia of corpus callosum	[90]	N/A	

CC, colon cancer; CRCs, colorectal cancers; GC, gastric cancer; N/A, not applicable/reported; OC, ovarian cancer; PCa, prostate cancer; Ref., reference.

**Table 2:** The molecular mechanism of MCPH-associated genes relative to neurogenesis and carcinogenesis.

Gene	Neurogenesis	Ref	Carcinogenesis	Ref.
*MCPH1 (BRIT1)*	CHK1, CDC25, CDK1	[16]	SLUG, CDK1, p53, CDH1, MDM2, SNAIL	[42]
			p53	[110]
			CyclinA2, CDK2, CDC25C-cyclinB, CDC2, p53, BCL-2, Bax, Cytochrome c, Caspase-3, PARP-1	[40]
*MCPH2* *(WDR62)*	JNK	[18]	N/A	
	AURKA	[119]		
	PLK1	[43]		
*MCPH3 (CDK5RAP2)*	p35	[120]	N/A	
	MST1, TAZ (**Hippo**)	[121]		
*MCPH4 (CASC5)*	BUB1, BUBR, ZWINT-1	[122]	BUB1	[52]
			miR-193b-3p	[27]
			p53	[123]
*MCPH5* (*ASPM*)	**Wnt** p53	[21] [114]	**Wnt**	[55,124,125]
*MCPH6* *(CENPJ)*	KIF2A	[56]	N/A	
	ASCL1	[57]		
*MCPH7* *(STIL)*	CPAP	[58]	Casepase-3/7, MAPK/ERK, PI3K/AKT, AMPK	[28]
	RTTN	[60]	PD-L1	[126]
	CDK1	[104]	IGF-1/PI3K/Akt	[32]
	GLI1(**Shh**)	[127]	GLI1(**Shh**)	[128]
*MCPH10 (ZNF335)*	REST/NRSF	[65]	N/A	
*MCPH12* *(CDK6)*	GLI3 (**Shh**)	[66]	FOXO3	[67]
	Neuroglobin	[70]	GLI2 (**Shh**)	[129]
	p27	[68]	p16INK4a, VEGF-A	[71]
			CD25, **Notch**	[72]
			SUMO1	[69]
*MCPH13* *(CENPE)*	N/A		FOXM1	[25]
*MCPH17* *(CIT)*	Tubulin β-III	[76]	p53	[26]
	Trp53	[77]	Tp53, Tp73	[33]
*MCPH18* *(WDFY3)*	**Wnt**	[79]	miR-18a, RORA	[78]
*MCPH19* *(COPB2)*	Camk1γb	[130]	p21, WAF1/CIP1, P27 KIP1, CDK2, CDK4, Cyclin D1	[82]
			YAP1 (**Hippo**)	[83]
			RTK	[86]
*MCPH20* *(KIF14)*	N/A		p27 (KIP1), SKP2, CKS1	[88]
			GADD45A, GADD45B, P21, PIDD, SHISA5, p53, **Hippo**	[36]
			AKT	[131]
			SP1, YY1	[87]
*MCPH21* *(NCAPD2)*	N/A		p53	[39]
*MCPH24* *(NUP37)*	N/A		YAP (**Hippo**)	[37]
*MCPH25* *(MAP11)*	PLK1	[90]	N/A	

N/A, not applicable/reported.

## Abbreviations

MCPH	MCPH Autosomal recessive primary microcephaly
NSC	Neural stem cell
CSCs	Cancer stem cells
NPCs	Neural progenitor cells
KO	Knockout
BrdU	5-bromo-2′-deoxyuridine
CCK-8	Cell counting kit-8
EdU	5-ethynyl-2′-deoxyuridine
FACS	Fluorescent-activated cell sorting
MTT	3-(4,5-dimethylthiazol-2-yl)-2,5-diphenyltetrazolium bromide
TUNEL	Terminal deoxynucleotidyl transferase (TdT)-mediated dUTP nick end labeling
HC	Head circumference
GC	Gastric cancer
OC	Ovarian cancer
CRCs	Colorectal cancers
CC	Colon cancer
PCa	Prostate cancer
CDKs	Cyclin-dependent kinases
GLI	Glioblastoma protein
FOXM1	Forkhead box M1
TP53	The p53 gene
pY15-Cdk1	Tyr 15-phosphorylated Cdk1
Cdk1b	Cyclin-dependent kinase inhibitor 1B
Shh	Sonic hedgehog
SUFU	Suppressor of fused
